# MEIS1 promotes expression of stem cell markers in esophageal squamous cell carcinoma

**DOI:** 10.1186/s12885-020-07307-0

**Published:** 2020-08-20

**Authors:** Selma Zargari, Shabnam Negahban Khameneh, Abolfazl Rad, Mohammad Mahdi Forghanifard

**Affiliations:** 1 Medical Genetics Research Center, Mashhad University of Medical Sciences, Mashhad, Iran; 2 Department of Biology, Damghan branch, Islamic Azad University, P.O.Box: 3671639998, Cheshmeh-Ali Boulevard, Sa’dei Square, Damghan, Islamic Republic of Iran; 3 Cellular and Molecular Research center, Sabzevar University of Medical Sciences, Sabzevar, Iran

**Keywords:** Self-renewal, ESCC, KYSE-30, *MEIS1*, Stemness markers

## Abstract

**Background:**

MEIS1 (Myeloid ecotropic viral integration site 1) as a homeobox (HOX) transcription factor plays regulatory roles in a variety of cellular processes including development, differentiation, survival, apoptosis and hematopoiesis, as well as stem cell regulation. Few studies have established pluripotency and self-renewal regulatory roles for MEIS1 in human esophageal squamous cell carcinoma (ESCC), and our aim in this study was to evaluate the functional correlation between MEIS1 and the stemness markers in ESCC patients and cell line KYSE-30.

**Methods:**

Expression pattern of *MEIS1* and *SALL4* gene expression was analyzed in different pathological features of ESCC patients. shRNA in retroviral vector was used for constantly silencing of *MEIS1* mRNA in ESCC line (KYSE-30). Knockdown of *MEIS1* gene and the expression pattern of selected stemness markers including *SALL4, OCT4, BMI-1, HIWI, NANOG, PLK1,* and *KLF4* were evaluated using real-time PCR.

**Results:**

Significant correlations were observed between MEIS1 and stemness marker SALL4 in different early pathological features of ESCC including non-invaded tumors, and the tumors with primary stages of progression. Retroviral knockdown of *MEIS1* in KYSE-30 cells caused a noteworthy underexpression of both *MEIS1* and major involved markers in stemness state of the cells including *SALL4, OCT4, BMI-1, HIWI* and *KLF4*.

**Conclusions:**

The results highlight the important potential role of *MEIS1* in modulating stemness properties of ESCCs and cells KYSE-30. These findings may confirm the linkage between *MEIS1* and self-renewal capacity in ESCC and support probable oncogenic role for MEIS1 in the disease.

## Background

Human esophageal cancer is the sixth leading cause of cancer-related mortality worldwide [1]. Esophageal squamous cell carcinoma (ESCC) is one of the main subtypes of esophageal cancer. In spite of using modern surgical techniques combined with adjuvant treatment, the overall 5-year survival rate of the patients still remains nearly 15–20% [2].

Increasing evidence demonstrate that tumors are maintained by cancer stem-like cells (CSCs). CSCs are a small population of cells with self-renewal capacity in most tumors which can promote tumor proliferation, metastasis, and drug resistance. CSCs are also considered as a source of cancer recurrence even after conventional therapies [3]. The two most essential properties of stem cells are pluripotency and self-renewal. Pluripotency is the capacity of stem cells to produce any cell type with specialized properties whereas the ability of self-renewal is described as the proliferation capacity of the cells for prolonged periods of time and regenerating the tissue [4].

Homeobox (*HOX*) genes, a large and essential family of developmental regulators, are vital for growth, differentiation and development of numerous organ systems [5, 6]. Myeloid ecotropic insertion site 1 (*MEIS1*) is a developmentally conserved member of 3-amino-acid loop extension (TALE) family which can interact with HOX proteins as a cofactor [7]. HOX and MEIS1 are involved in different biological processes such as chromatin remodeling, cell cycle control, apoptosis and differentiation, as well as transcription adjustment of self-renewal genes [8–10]. Deregulated *MEIS1* mRNA and protein expression can lead to tumorigenesis in a number of tumor types such as acute myeloid leukemia [11], lung adenocarcinoma tumors [12], neuroblastomas [13], ovarian carcinomas [14] and ESCC [15]. Recent evidence suggested a tight association between *MEIS1* and self-renewal signature in hematopoietic and neural stem cells [16]. Moreover, the correlation between *MEIS1* and CSC marker SOX2 has been shown in ESCC predicting cancer stemness properties for *MEIS1* in the disease [15]. Several CSCs markers are proposed as ESCC CSC markers such as OCT4, BMI-1, SALL4, HIWI and KLF4. Since these markers are frequently up-regulated in different malignancies, a regulatory role in maintenance of pluripotency and self-renewal has been suggested for these genes [17–22].

Since, phenotypic and functional properties of CSCs are regulated through a variety of extrinsic signaling pathways and intrinsic self-renewal factors [23–25], there is an urgent need to explore its details to provide specific targeted therapies for various cancers including ESCC. In the present study the correlation between expression pattern of MEIS1 and different stem cell markers including SALL4, OCT4, BMI-1, KLF4 and HIWI was investigated in ESCC patients and cell line to evaluate the potential correlation between *MEIS1* and stemness state of the cells.

## Methods

### Study population

The clinicopathological features of 50 ESCC patients and related gene expression pattern of MEIS1 and SALL4 in the tumors compared to the adjacent tumor free tissues were used in this study. As previously described [15, 26], patients selection was restricted to a specific conditions, and both informed consent of patients to be involved in the study and approval of the ethic committee of Mashhad University of Medical Sciences, Mashhad, Iran, were recorded.

### Cell lines and culture condition

Human ESCC (KYSE-30) and embryonic kidney (HEK293T) cell lines were purchased from the Pasteur Institute Cell Bank of Iran (http://en.pasteur.ac.ir/) and grown in RPMI 1640 medium (Biosera) and Dulbecco’s modified Eagle’s medium (DMEM; Biosera), respectively. Both culture media were supplemented with 10% heat-inactivated fetal bovine serum (FBS; Gibco, USA), 100 U/ml, and 100 μg/ml penicillin-streptomycin (Gibco, USA) and cultured at a humidified atmosphere 37 ͦ C with 5% CO2. The KYSE-30 cell line was last successfully authenticated by short tandem repeat profiling at the Pasteur Institute Cell Bank of Iran.

### *MEIS1* gene expression knockdown

Based on principles of shRNA design and the human *MEIS1* structure (GenBank reference sequence: NM_002398.3) [27], the lentivirus-based pLKO.1-puro plasmid (Cat. No. SHC003) was constructed by Sigma-Aldrich (St. Louis, MO). The pLKO.1-puro plasmid DNA was labeled with a cytomegalovirus (CMV) promoter driving expression of the green fluorescent protein (GFP) gene. Lentivirus production was followed by transfecting HEK293T cells according to the standard calcium phosphate method with pLKO.1-MEIS1, together with the psPAX2 and the pMD2.G as packaging vectors [28] (plasmids 12,260 and 12,259, respectively, Cambridge, MA). Viral supernatant was harvested 24 and 48 h after transfection, filtered through a 0.45-μm filter (Orange, Belgium). Then, the virus was recovered after ultracentrifugation (40-mL culture medium per 50-mL Beckman tube, ultracentrifugation 70,000×g, 4 °C for 2 h) and resuspended in fresh medium, and used to transduce KYSE-30 cells which were cultured at a density of 0.5–1 × 10^6 cells in 6-well plate the previous day. Cells were continuously cultured for 4 to 5 days followed by selection with puromycin (Invitrogen Corporation, Carlsbad, CA). The transduced KYSE-30 cells with recombinant lentiviral particles of GFP (control) and GFP-shMEIS1 were assayed using inverted fluorescence microscopy.

### RNA extraction, cDNA synthesis, comparative real time PCR

Tripure reagent (Roche, Nutley, NJ) was used to extract RNA from GFP and GFP-shMESI1 transduced ESCC cell line, as recommended by the manufacturer. Subsequently DNase I (Thermo Fisher Scientific, Waltham, MA) treatment was performed for preventing DNA contamination. Total RNA was used as a template for the synthesis of cDNA using the oligo-dT method (Fermentas, Lithuania). Following cDNA synthesis, qRT-PCR was used to assess *MEIS1* mRNA knockdown. Furthermore, relative comparative changes of *BMI1* (GenBank reference sequence: NM_005180.9), *SALL4* (GenBank reference sequence: NM_001318031.2), *KLF4* (GenBank reference sequence: NM_001314052.2), *OCT4* (GenBank reference sequence: NM_001173531.2), NANOG (GenBank reference sequence: NM_024865.4), PLK1 (GenBank reference sequence: NM_005030.6), and *HIWI* (GenBank reference sequence: NM_001190971.2) mRNA expressions were assessed in *MEIS1* silenced compared to GFP control cells using a relative comparative real-time PCR using gene-specific primer sets shown in Table 1. *GAPDH* housekeeping gene was used as a normalizer and 2-ΔΔCt method was used to measure fold changes of gene expression [29]. Briefly, PCR was performed in a total volume of 20 μL in 1 × SYBR Green Real Time PCR Master Mix (AMPLIQON, Denmark) containing 0.5 μM of each primer and was done on a LightCycler® 96 Real-Time PCR System thermocycler (Roche, Germany**).** While the log2 fold changes in mRNA expression more than 2, and less than − 2 folds were considered as overexpression and underexpression, respectively, the range in between was regarded as normal expression.

**Table 1:** Primer sequences used for qRT-PCR in this study

**Gene**	**Forward primer**	**Reverse primer**	**Amplicon size**
***MEIS1***	ATGACACGGCATCTACTCGTTC	TGTCCAAGCCATCACCTTGCT	105
***BMI1***	CGTGTATTGTTCGTTACCTGGAGAC	CATTGGCAGCATCAGCAGAAGG	204
***SALL4***	CCAGGGAATGACGAGGTGG	GAACTCCGCACAGCATTTCTC	96
***KLF4***	TCTTCTCTTCGTTGACTTTG	GCCAGCGGTTATTCGG	210
***OCT4***	GAACATGTGTAAGCTGCGGCC	CCCTTCTGGCGCCGGTTAC	148
***HIWI***	ATGATTGAAGTGGATGACAGAACTG	TACTTGACAACAGACAGACAACTAT	97
***GAPDH***	GGAAGGTGAAGGTCGGAGTCA	GTCATTGATGGCAACAATATCCACT	101
***NANOG***	GGCAATGGTGTGACGCAGAAGGC	GCTCCAGGTTGAATTGTTCCAGGTC	137
***PLK1***	ATAGAGCGTGACGGCACTGAGT	TGCTCGCTCATGTAATTGCG	107

### Statistical analysis

The SPSS 19.9 statistical package (SPSS, Chicago, IL, USA) was applied for statistical data analysis. *P* value < 0.05 was regarded as statistically significant. We used the χ2 or Fisher exact tests and Pearson’s correlation to evaluate the association between gene expressions.

## Results

### *MEIS1* gene expression is correlated with *SALL4* in ESCC patients

**Fig. 1:**
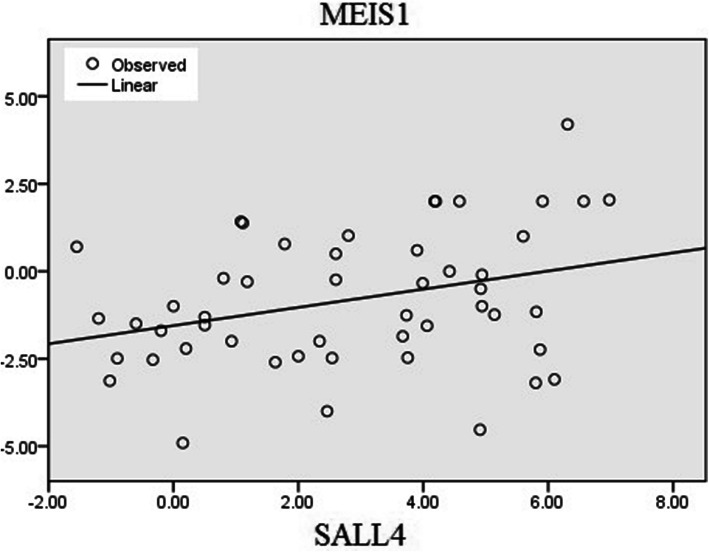
Correlation between mRNA expression of *MEIS1* and *SALL4* in ESCC patients is depicted as regression plot. Tumor samples with elevated level of *MEIS1* expression show a high level of *SALL4* expression as well (P = 0.022, correlation coefficient: 0.322). X and Y axis show log2 fold change of gene expression

Gene expression pattern of *MEIS1* and *SALL4* in 50 ESCC patients was used here to analyze correlation between these genes in different clinicopathological features of the patients. The clinicopathological characteristics of recruited patients are presented previously [15]. Based on statistical analysis, a significant correlation was observed between *MEIS1* and *SALL4* gene expression in ESCCs (*P* = 0.022, correlation coefficient: 0.322). The expression pattern of *MEIS1* and *SALL4* was synced to each other in more than half of the patients (52%, 26 of 50 samples). As described in Table 2, overexpression of both genes was observed in nine patients and concomitant unchanged/underexpression of the genes was detected in 17 tumor samples. Correlation between the genes is depicted in Fig. 1 as regression plot.

**Table 2:** Concomitant expression of *MEIS1* and *SALL4* in ESCCs (*P* = 0.022)

		*SALL4* expression		Total
		Normal/under	Overexpression	
*MEIS1* expression	Normal/under	17	22	39
	Over	2	9	11
Total		19	31	50

Interestingly, significant correlation was detected between *MEIS1* and *SALL4* in non-invaded tumors into the adventitia (T1/T2, *P* = 0.017, correlation coefficient: 0.699) but not in invaded tumors (T3/T4, *P* = 0.114, correlation coefficient: 0.257). In addition, *MEIS1* was significantly correlated with *SALL4* in tumors without metastasis to the lymph node (*P* = 0.023, correlation coefficient: 0.427) in contrast with lymph node metastasized tumors (*P* = 0.453, correlation coefficient: 0.169). And finally, a noteworthy correlation between the genes was found in early stages of tumor progression (stage I/II, *P* = 0.030, correlation coefficient: 0.390), while in advanced stages of the disease (stages III/IV) the correlation was not significant (*P* = 0.439, correlation coefficient: 0.189). The expression pattern of *MEIS1* and *SALL4 *in different pathological states of the ESCCs is summarized in Table 3.

**Table 3:** The expression pattern of *MEIS1* and *SALL4* in different pathological states of the ESCCs

		MEIS1 expression		SALL4 expression		*p*-value
		Normal/under	Over	Normal/under	Over	
Sex	Male	18	6	9	15	*P* = 0.521
	Female	21	5	10	16	
Lymph node metastasis	No metastasis	20	8	13	15	*P* = 0.023*
	Node metastasis	19	3	6	16	
Depth of invasion	T1,2	8	3	1	10	*P* = 0.017*
	T3,4	31	8	18	21	
Stage of progression	Stage I/II	23	8	12	19	*P* = 0.030*
	Stage III/IV	16	3	7	12	
Grade of differentiation	P.D**	5	1	4	2	*P* = 0.231
	M.D	27	6	9	24	
	W.D	7	4	6	5	
Location	Lower	18	4	9	13	*P* = 0.327
	Middle	20	6	9	17	
	Upper	1	1	1	1	

*Asterisk show statistical significance

**P. D Poorly Differentiated, M. D Moderatly Differentiated, W. D Well Differentiated

### Lentivirus-mediated shRNA efficiently knocks down expression of *MEIS1*

To deliver shRNA into the esophageal cancer cell line KYSE30, we used a lentiviral-based vector that expressed *MEIS1* shRNA. KYSE30 cells were transduced with *MEIS1* expressing viral particles and selected by puromycin 48 h after transduction. Ten days post transduction; cells were analyzed for *MEIS1* expression using real-time PCR. Compared with the negative control group the level of *MEIS1* (mRNA) expression in the infected cells was sharply reduced (log2 fold change: − 5.6). These data demonstrated that the expression of MEIS1 gene is efficiently downregulated in transduced cells KYSE-30.

### Down-regulated expression of *MEIS1* by shRNA decreased the expression of stemness genes

**Fig. 2:**
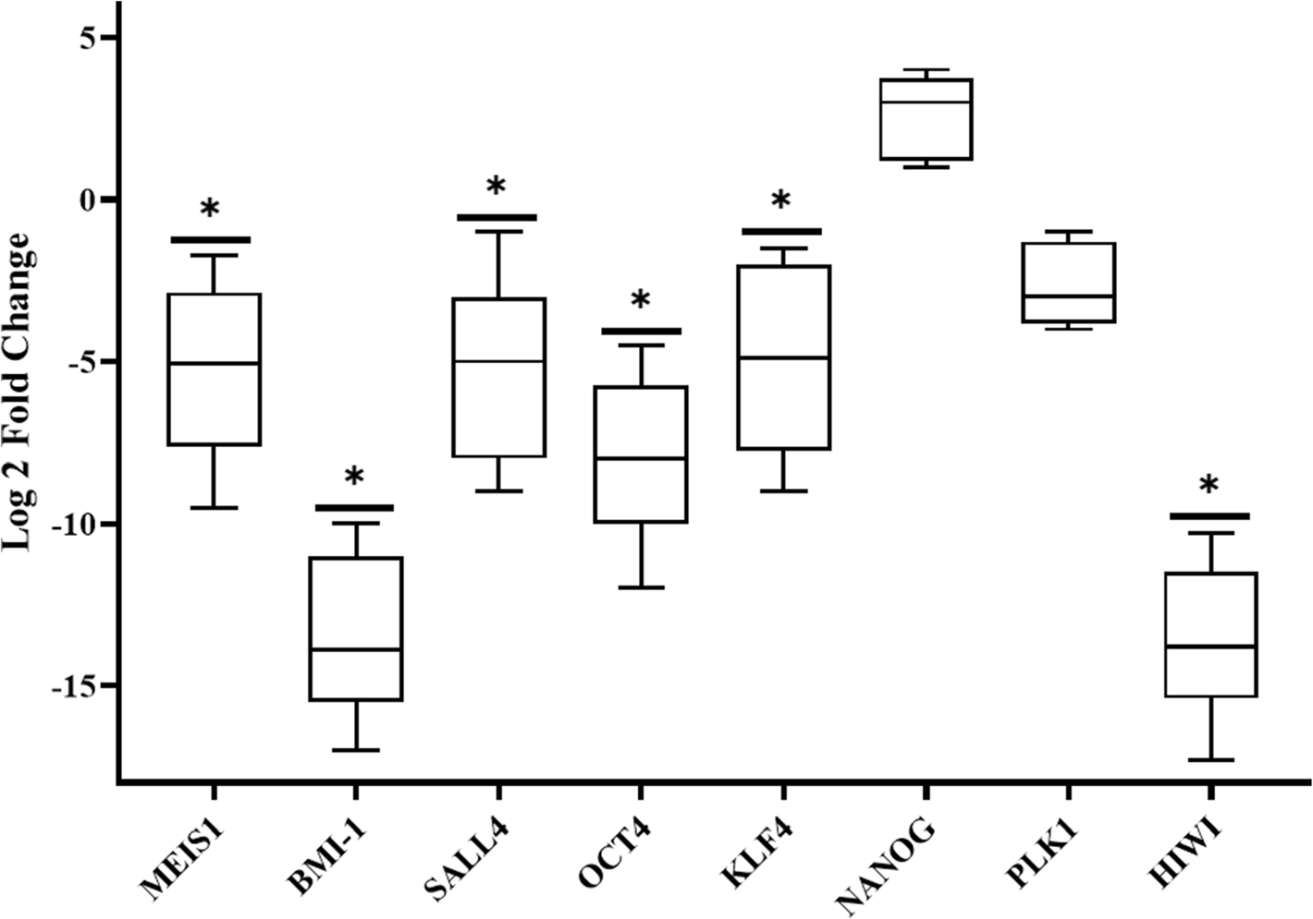
mRNA expression Levels of *MEIS1* and different stem cell markers in MEIS1-silenced cells compared to control are represented as box plots. Each box plot shows median, lower/upper quartile, and highest/lowest observations of log2 fold changes. Asterisks mean statistically significance (*P* ≤ 0.05)

The expression of cancer stem cell markers was assessed in *MEIS1* silenced cell line compared to control. Downregulation of *MEIS1* led to a significant decrease in the levels of the most important stem cell markers *BMI1*, *SALL4, OCT4* and *KLF4* mRNA expression (log2 fold change: − 14.28, − 5, − 7.14 and − 5.26 fold, respectively) in KYSE30 cells. Furthermore, the level of *HIWI* mRNA expression was significantly reduced about − 14.28 in *MEIS1* silenced cells in comparison with control. The levels of gene expression are presented in Fig. 2 as box plot. These data clearly showed the significant decrease in expression of the majority of selected stemness genes in KYSE-30 cells after *MEIS1* silencing. No changes were observed in mRNA expression of *NANOG* and *PLK1* following silencing of *MEIS1* in KYSE-30 cells.

## Discussion

ESCC is one of the invasive malignancies of gastrointestinal tract with considerable mortality and morbidity rate [30]. Therefore, understanding the molecular mechanisms behind the esophageal tumorigenesis is crucial for achieving the best diagnostic and therapeutic approaches. Different cancer propagation models have been described and CSC model is an interesting one. However, evaluating the stemness behavior of ESCC has not been widely studied.

The present study demonstrated the impact of *MEIS1* on expression of stem cell markers in ESCC and found that mRNA expression of major stem cell markers including SALL4, OCT4, BMI-1, HIWI and KLF4 was significantly decreased in *MEIS1* silenced cells compared to control. Furthermore, the expression patterns of *MEIS1* and stemness marker *SALL4 *were significantly associated to each other depending on different pathological features of the patients, specifically in early stages of tumor progression.

The correlation between *MEIS1* and involved genes in self-renewal and pluripotency of different CSCs has been discussed in few studies. It has been revealed that meis1^−/−^ mice die because of abnormalities in hematopoiesis and vascularization due to lack of hematopoetic stem cell (HSC) niches in the embryos. These findings highlighted the important role of MEIS1 in HSC regulation [31, 32]. MEIS1 has a critical role in cardiomyocyte proliferation and HSC expansion as well as regulation of cellular metabolism [33]. Besides the role of MEIS1 in healthy organs, maintaining stemness state of cancer stem cells has been also discussed in various cancers. In some cancers including MLL fusion leukemia, it has been reported that MEIS1 is crucial for maintenance of the stem cell molecular profile [34]. Using a knock-in model of mouse leukemia (MLL-AF9), it has been demonstrated that MEIS1 is necessary for maintaining an ESC-like gene signature [34]. In other cancers including neuroblastoma, high level expression of *MEIS1* and *MEIS2* genes was demonstrated, and defective *MEIS1* cells showed impaired proliferation leading to cell death [13].

We have recently reported that MEIS1 knockdown in KYSE-30 cells can induce expression of epithelial differentiation markers CDX2, and KRT4, while it can suppress the involved genes in EMT process including TWIST1, EGF [35]. In line with this report, our presented results in this study support the potential oncogenic role for MEIS1 in promoting mesenchymal/stemness phenotype of ESCC. The role of *MEIS1* and its correlation with *SOX2* in ESCC has been previously evaluated [15]. *MEIS1* expression is decreased in ESCC and inversely related to lymph node metastasis and high tumor stage. Moreover, down regulation of *MEIS* was correlated with increased expression of *SOX2*, a master transcription factor of self-renewal [15]. In the present study we evaluated the expression of some CSC markers in ESCC after silencing of *MEIS1* and a similar pattern was detected. Various CSCs markers including SALL4, OCT4, BMI-1, HIWI and KLF4 were underexpressed after MEIS1 downregulation. These markers have been proposed as potential self-renewal markers associated with aggressiveness, poor prognosis and cancer recurrence in ESCC [36, 37]. Interestingly, expression of MEIS1 and SALL4 was correlated to each other in ESCCs. Having split the patients based on different pathological states of tumors, we found significant correlation between the genes in primary steps of tumor growth. Indeed, these correlations were observed in tumors without invasion to adventitia layer of the esophagus (T1 and T2) presenting early stages of carcinogenesis (stages I and II). This observation may propose a functional involvement of the genes in beginning and promoting ESCC carcinogenesis through advanced stages. Furthermore, a correlation of MEIS1 and SALL4 was found in tumor samples without metastasis, compared to metastasized ESCCs. This correlation indicates a contribution of MEIS1/SALL4 expression in operating cancer aggressiveness in ESCC.

*OCT4*, *KLF4* and *SALL4* are a members of a core regulatory network of stem cell maintenance and self-renewal [38]. The relation between these key stemness factors has been addressed in the literature. The complexity and relation of these stemness factors can be seen in various cancers [39–41]. Also, it has been reported that downregulation of *SALL4* resulted in downregulation of both *OCT4* and *KLF4*, ending up in decreasing in reprogramming capacity to induce pluripotent stem cells [42].

In our experience, following MEIS1 down-regulation in ESCC, expression of SALL4 was reduced significantly. Aberrant expression of *SALL4* observed in different types of cancers and disruption of multiple cellular tumorigenesis processes suggested a key stemness regulatory effect for *SALL4* [43–46]. The possible linkage between *SALL4* and other genes discussed in the present study was suggested previously. The role of *SALL4*, as a major regulator of pluripotency in stem cells, was evaluated in murine-embryonic stem cells and demonstrated that *SALL4* downregulation decreased *KLF4* expression; the proteins involved in reprogramming somatic cells to pluripotent cells [42].

OCT4 as a critical transcription factor and stem cell marker, is only activated during human embryonic development in pluripotent stem cells, and its expression decreases after stem cell fate decision during embryogenesis [47, 48]. Our results demonstrated that expression of *OCT4* is reduced in *MEIS1* silenced ESCC. Yamada et al. demonstrated that MEIS1 is tightly associated with self-renewal signature in hematopoietic and neural stem cells, and can regulate the transcription of the critical stemness genes including *OCT4,* in such cells [16]. While OCT4 is absent in normal human adult tissue, it has been demonstrated that some benign and malignant human tumors can express *OCT4* [49]. OCT4 is a key stemness transcription factor [21, 42, 50, 51] and the importance of *OCT4* during carcinogenesis is becoming more evident. Recently, Kim et al. demonstrated that *OCT4* expression plays a crucial role in inducing pluripotency in adult neural stem cells, alongside with other markers including SOX2, c-Myc, and KLF4 [17]. *OCT4* is regulated by a well-known protein, SALL4. It has been demonstrated that SALL4 can bind to *OCT4* promoter and modulate its expression [52, 53]. Furthermore, expression of BMI-1 was also decreased after MEIS1 knockdown in KYSE-30 cells. It has been demonstrated that SALL4 can upregulates the oncogene *BMI-1* expression in human hematopoietic stem cells as well as leukemic cells [54]. *BMI-1* is overexpressed in aggressive and recurrent tumors and regulates proliferation, differentiation and senescence of the cells [55]. Increased levels of BMI-1 activated the stemness state in gastric cancer cells, induced by overexpression of *SALL4* [56]. Based on the mentioned evidences and our results, the correlation between MEIS1 and BMI-1 may be mediated by SALL4.

*KLF4*, member of the Kruppel-like factor (KLFs) family of gene regulatory proteins, implicated in the regulation of cell-fate, differentiation, and migration, as well as cancer metastasis [57, 58]. Reprogramming of somatic cells into pluripotent cells is another major role of *KLF4* [19, 20, 59, 60]. According to our results, *KLF4* expression was reduced after *MEIS1* silencing in ESCC.

Similar to *OCT4* and *SALL4*, the expression of *HIWI* was reduced after silencing of *MEIS1* in ESCC line KYSE-30. While HIWI is a self-renewal marker dealing with regulation of stem cell self-renewal and maintenance [22, 61, 62], it’s overexpression caused tumorigenesis in multiple malignancies and plays a specific role in CSC-like characteristics of cancer cells [63]. The upregulation of *HIWI* is significantly associated with a higher clinical stage, and a poorer clinical outcome in esophageal cancer cells. Our study revealed that the level of *HIWI* mRNA expression was significantly decreased in *MESI1* silenced cells in comparison with control cells. This finding has not been widely studied before and the contribution of *MEIS1* in *HIWI* gene regulation should be explored in detail.

Collectively, our results present evidences supporting oncogenic roles for MEIS1 in ESCC through correlation with different stem cell markers.

## Conclusions

The present study demonstrated the important role of *MEIS1* in controlling stemness properties of ESCC line KYSE-30. Here we elucidated the correlation between MEIS1 and stemness marker SALL4 in ESCC and revealed significant correlation between the genes in different early pathological features of the disease including non-invaded state, at primary stages of tumor progression. Furthermore, we demonstrated that expression of certain stemness factors including *SALL4*, *OCT4*, *BMI-1*, *HIWI* and *KLF4* genes were significantly decreased after *MEIS1* silencing in ESCC line KYSE-30. To the best of our knowledge, this is the first report highlighting the linkage between *MEIS1* and the major markers involving in stemness and self-renewal maintenance. These findings suggest a possible therapeutic role for *MEIS1* in future cancer therapies based on targeting self-renewal capacities of cancer cells in ESCC.

## Data Availability

All raw data are available in case of request.

## Abbreviations

MEIS1: Myeloid ecotropic viral integration site 1
HOX: Homeobox
ESCC: Esophageal Squamous Cell Carcinoma
SALL4: Sal-like protein 4
OCT4: Octamer-binding Transcription Factor 4
BMI-1: B cell-specific Moloney Murine leukemia Virus Integration Site 1
HIWI: Piwi Like RNA-Mediated Gene Silencing 1
PLK1: Polo Like Kinase 1
KLF4: Kruppel Like Factor 1
TALE: 3-amino-acid Loop Extension
TWIST1: Twist Family BHLH Transcription Factor 1
EGF: Epidermal Growth Factor
CDX2: Caudal Type Homeobox 2
KRT4: Keratin 4
EMT: Epithelial-Mesenchymal Transition
CSC: Cancer Stem-like Cells
HSC: Hematopoetic Stem Cell
ShRNA: short hairpin RNA
GAPDH: Glyceraldehyde 3-Phosphate Dehydrogenase
CMV: Cytomegalovirus
GFP: Green Fluorescent Protein
